# Supplementary material for: ASIC1a is required for neuronal activation via low-intensity ultrasound stimulation in mouse brain
Source: eLife. 2021 Sep 27;10:e61660. doi: 10.7554/eLife.61660 (PMC8510583; doi:10.7554/eLife.61660)
Supplement: Supplementary file 1. [file elife-61660-supp1.docx]

ASIC1a is required for neuronal activation via low-intensity ultrasound stimulation in mouse brain

Jormay Lim, Hsiao-Hsin Tai, Wei-Hao Liao, Ya-Cherng Chu, Chen-Ming Hao, Cheng-Han Lee, Shao-Shien Lin, Sherry Hsu, Ya-Chih Chien, Dar-Ming Lai, Wen-Shiang Chen, Chih-Cheng Chen and Jaw-Lin Wang

Jormay Lim, Hsiao-Hsin Tai and Wei-Hao Liao contribute equally.

Address correspondence and requests for reprints to

Jaw-Lin Wang, Ph.D., corresponding author

Professor, Department of Biomedical Engineering, College of Medicine and College of Engineering, National Taiwan University,

Address: 602 Jen-Su Hall, 1 Section 4, Roosevelt Road, Taipei 10617, Taiwan, ROC

Phone: 886-2-33665269, Fax: 886-2-23687573, Email: [jlwang@ntu.edu.tw](mailto:jlwang@ntu.edu.tw)

Chih-Cheng Chen, Ph.D. corresponding author

Research fellow, Institute of Biomedical Sciences, Academia Sinica

Address: 128 Academia Road, Section 2, Taipei 115, Taiwan

Phone: 886-2-26523917, Fax: 886-2-27829224, Email: chih@ibms.sinica.edu.tw

Supplementary file 1A: In vivo animal and human transcranial ultrasound experiments.

| Reference | Disease & Model | Ultrasound parameters | Finding |
| --- | --- | --- | --- |
| Liu 2019 (1) | Acute ischemic stroke, In vivo rat model | 0.5 MHz, Isppa=2.6 W/cm2, (Ispta=173 mW/cm2), Dose: 10 min/day | Ultrasound increase the Apparent Diffusion Coefficient (ADC) of MRI, the earlier intervention (0.5 h) is better than late (up to 9 h).  Rationale: US increase fluid flow within the brain |
| Eguchi 2018 (2) | Vascular & Alzheimer’s Dementia, In vivo mice model | 1.875 MHz, DF=10%, Ispta=90 mW/cm2, Dose: 20 min *3 days | LIPUS improved cognitive dysfunctions due to eNOS expression.  Rationale: US increase the vascular endothelial cell eNOS. |
| Sato 2016 (3) | Inferior alveolar nerve (IAN) injury, In vivo rat model | 1 MHz, Isata=30 mW/cm2, Dose: 20 min/day for 28 d | Increased mechanical sensitivity, and TG cell number.  Rationale: US increase trigeminal ganglion (TG) cell number. |
| Baek 2018 (4) | Cerebellar ischemic stroke, MCAO model, in vivo mice model | 0.35 MHz, Isppa=2.54 W/cm2, (Ispta=127 mW/cm2), Dose: 20 min/day for 2 d | Increased ipsilateral water content due to tissue swelling, showing attenuation of brain edema. Prominently, the reduction of neuro-immune reactivity at the infarct core and peri-infarct region. |
| Lin 2015 (5) | Al-induced Alzheimer dementia,  In vivo Rat model | 1 MHz, DF=5%, Ispta=528 mw/cm2, Dose: 5 min*3/day | Increase BDNF, GDNF, VEGF, Decrease Al particle, acetylcholinesterase, beta-amyloid, karyopyknosis, Increased behavioral test.  Rationale: US increase BDNF |
| Hung 2017 (6) | Bilateral common carotid artery occlusion (BCCAO) induced vascular dementia | Same as above, | Increased BDNF, myelin using micro-PET images, and histology  Recovered hippocampus neuron, increased behavioral test  Rationale: US increase BDNF |
| Su 2017 (7),  Chen 2018 (8),  Su 2017 (9) | Cortical impact injury induced brain trauma | Same as above, | Reduced brain edema, blood brain barrier permeability, and neuronal degeneration at day 1, improved functional recovery and reduced contusion volume at day 28. At day 4 reduced MMP9, increased BDNF, enhance p-TrkB, Akt, cAMP response  Rationale: US increase BDNF |
| Chen 2018 (10) | Cerebral ischemia/reperfusion injury using middle cerebral artery occlusion (MCAO) model | Same as above | Apoptosis reduction and BDNF induction in MCAO model  Rationale: US increase BDNF |
| Chen 2019 (11) | Lipopolysaccharide (LPS) induced Alzheimer’s | Same as above | Increased behavioral test, Decreased beta-amyloid, APP, Caspase-3, GFAP, TNF-alpha, IL-1beta, IL-6 at hippocampus, and cortex, NF-kapaB, TLR4 signal pathway, Increased BDNF and CREB  Rationale: US increase BDNF |
| Lin 2018 (12) | Brain glioblastoma | 1 MHz, A392S transducer Energy= 2.86 W, DF=5%, Stimulation: 60 sec | US as a BBB disruption, increased Dox concentration |
| Tyler 2008 (13) | mice brain slice (400 micron) | 0.44 MHz, Isppa=2.9 W/cm2, Ispta=23 mW/cm2, DF=1%, P=0.8MPa, | US regulate voltage-gated sodium and calcium channels. Trigger SNARE-mediated exocytosis and synaptic transmission. |
| Nicodemus 2019 (14) | Clinical trial of focused US for AD | Ispta=520 mW/cm2, DF=0.1%, P= 5 MPa, Stimulation=1 hour | Cognition: 1/3 improved, 1/3 decreased, 1/3 remain same  Motor: 5% improved, 15% decreased,  MRI showed immediate increased blood flow perfusion. |
| Legon 2014 (15),  Panczykowski 2014 (16) | Human cortical | 0.5 MHz, Isata=4.295 W/cm2, DF=36%, P=0.8MPa, Stimulation=0.5 sec | Ultrasound can focally modulate cortical function, indicated by changed alpha, beta, gamma wave function |

**References**

1. L. Liu *et al.*, Protective effect of low-intensity transcranial ultrasound stimulation after differing delay following an acute ischemic stroke. *Brain Res Bull* **146**, 22-27 (2019).

2. K. Eguchi *et al.*, Whole-brain low-intensity pulsed ultrasound therapy markedly improves cognitive dysfunctions in mouse models of dementia - Crucial roles of endothelial nitric oxide synthase. *Brain Stimul* **11**, 959-973 (2018).

3. M. Sato, M. Motoyoshi, M. Shinoda, K. Iwata, N. Shimizu, Low-intensity pulsed ultrasound accelerates nerve regeneration following inferior alveolar nerve transection in rats. *Eur J Oral Sci* **124**, 246-250 (2016).

4. H. Baek *et al.*, A neuroprotective brain stimulation for vulnerable cerebellar Purkinje cell after ischemic stroke: a study with low-intensity focused ultrasound. *Conf Proc IEEE Eng Med Biol Soc* **2018**, 4744-4747 (2018).

5. W. T. Lin, R. C. Chen, W. W. Lu, S. H. Liu, F. Y. Yang, Protective effects of low-intensity pulsed ultrasound on aluminum-induced cerebral damage in Alzheimer's disease rat model. *Sci Rep* **5**, 9671 (2015).

6. S. L. Huang, C. W. Chang, Y. H. Lee, F. Y. Yang, Protective Effect of Low-Intensity Pulsed Ultrasound on Memory Impairment and Brain Damage in a Rat Model of Vascular Dementia. *Radiology* **282**, 113-122 (2017).

7. W. S. Su, C. H. Wu, S. F. Chen, F. Y. Yang, Low-intensity pulsed ultrasound improves behavioral and histological outcomes after experimental traumatic brain injury. *Sci Rep* **7**, 15524 (2017).

8. S. F. Chen, W. S. Su, C. H. Wu, T. H. Lan, F. Y. Yang, Transcranial Ultrasound Stimulation Improves Long-Term Functional Outcomes and Protects Against Brain Damage in Traumatic Brain Injury. *Mol Neurobiol* **55**, 7079-7089 (2018).

9. W. S. Su, C. H. Wu, S. F. Chen, F. Y. Yang, Transcranial ultrasound stimulation promotes brain-derived neurotrophic factor and reduces apoptosis in a mouse model of traumatic brain injury. *Brain Stimul* **10**, 1032-1041 (2017).

10. C. M. Chen, C. T. Wu, T. H. Yang, S. H. Liu, F. Y. Yang, Preventive Effect of Low Intensity Pulsed Ultrasound against Experimental Cerebral Ischemia/Reperfusion Injury via Apoptosis Reduction and Brain-derived Neurotrophic Factor Induction. *Sci Rep* **8**, 5568 (2018).

11. T. T. Chen, T. H. Lan, F. Y. Yang, Low-Intensity Pulsed Ultrasound Attenuates LPS-Induced Neuroinflammation and Memory Impairment by Modulation of TLR4/NF-kappaB Signaling and CREB/BDNF Expression. *Cereb Cortex* **29**, 1430-1438 (2019).

12. Y. L. Lin, M. T. Wu, F. Y. Yang, Pharmacokinetics of doxorubicin in glioblastoma multiforme following ultrasound-Induced blood-brain barrier disruption as determined by microdialysis. *J Pharm Biomed Anal* **149**, 482-487 (2018).

13. W. J. Tyler *et al.*, Remote excitation of neuronal circuits using low-intensity, low-frequency ultrasound. *Plos One* **3**, e3511 (2008).

14. N. E. Nicodemus *et al.*, Focused transcranial ultrasound for treatment of neurodegenerative dementia. *Alzheimers Dement (N Y)* **5**, 374-381 (2019).

15. W. Legon *et al.*, Transcranial focused ultrasound modulates the activity of primary somatosensory cortex in humans. *Nat Neurosci* **17**, 322-329 (2014).

16. D. M. Panczykowski, E. A. Monaco, 3rd, R. M. Friedlander, Transcranial focused ultrasound modulates the activity of primary somatosensory cortex in humans. *Neurosurgery* **74**, N8 (2014).

Supplementary file 1B: Immunohistochemical staining of p-ERK expression in Control and Ultrasound treated wild type mouse brain.

| Brain region (Bregma:-1.5,-2) | WT Control | Ultrasound |
| --- | --- | --- |
| **Cortex (Posterior parietal association areas)** | **-** | **+** |
| **Cortex (Motor area)** | **-** | **-** |
| - Secondary motor area | **-** | **-** |
| - Primary motor area | **-** | **-** |
| **Cortex (Somatosensory area)** | **+** | **+** |
| - Primary somatosensory area, trunk | **-** | **+** |
| - Primary somatosensory area, barrel | **-** | **+** |
| **Cortex (Auditory area)** | **-** | **+** |
| - Dorsal auditory area | **-** | **+** |
| - Primary auditory area | **-** | **+** |
| - Ventral auditory area | **-** | **+** |
| - Temporal association areas | **-** | **+** |
| **Cortex (Entorhinal area)** | **+** | **++** |
| **Endopiriform nucleus** | **+** | **-** |
| **Caudoputamen** | **-** | **+** |
| **Hippocampus (CA1 subfield)** | **-** | **-** |
| **Hippocampus (CA2 subfield)** | **-** | **-** |
| **Hippocampus (CA3 subfield)** | **-** | **-** |
| **Hippocampus (dentate gyrus)** | **-** | **-** |
| **Central Amygdalar nucleus** | **-** | **+++** |
| **Basolateral Amygdalar nucleus** | **+** | **+** |
| **Cortical Amygdalar nucleus** | **-** | **+** |
| **Medial Amygdalar nucleus** | **-** | **+** |
| **Piriform cortex** | **+** | **++** |

| Brain region (Bregma:-2.7,-3) | WT Control | Ultrasound |
| --- | --- | --- |
| **Retrosplenial area** | **-** | **+** |
| **Cortex (Visual area)** | **-** | **+** |
| - Anteromedial visual area | **-** | **+** |
| - Primary visual area | **-** | **+** |
| - Amterolateral visual area | **-** | **+** |
| **Cortex (Posterior parietal association areas)** | **+** | **+** |
| **Cortex (Auditory area)** | **-** | **++** |
| - **Dorsal auditory area** | **-** | **+** |
| - **Primary auditory area** | **-** | **++** |
| - **Temporal association areas** | **-** | **+** |
| **Cortex (Ectorhinal area)** | **-** | **++** |
| **Cortex (Piriform area)** | **-** | **-** |
| **Cortex (Entorhinal area)** | **-** | **+** |
| **Cortex (Perirhinal area)** | **-** | **+** |
| **Postpiriform transition area** | **++** | **+** |
| **Cortical Amygdalar area** | **-** | **-** |
| **Hippocampus (CA1 subfield)** | **+** | **++** |
| **Hippocampus (CA3 subfield)** | **-** | **-** |
| **Hippocampus (dentate gyrus)** | **+** | **++** |
| **Superior colliculus** | **-** | **+** |
| **Subiculum** | **-** | **+** |

The sections were incubated with anti-p-ERK antibody and stained with avidin–biotin-peroxidase for visualization. p-ERK expression was quantified by the number of positive staining cells was calculated using optical microscopy in 100 μm coronal brain sections from control and ultrasound treated wild type mice brain. ‘‘-’’ absent; ‘‘**+**’’: low expression; ‘‘**++**’’ : moderate expression; ‘‘**+++**’’: high expression

Tables summarize results of 2-way ANOVA analysis:

Supplementary file 1C: Two-way ANOVA analysis of the numbers of AUC data points listed in the bar charts comparing the calcium response of CHO cells stimulated by micropipette guided ultrasound (Figure 4B).

| **Area under curve (AUC)**  **Source of variation** | **CHO cells calcium response** | |
| --- | --- | --- |
|  | **F value** | **p value** |
| **Interaction** | 2.085 | **0.086** |
| **ASIC1a overexpression** | 8.32 | **<0.0001** |
| **Ultrasound**  **treatment** | 7.67 | **0.0083** |

Supplementary file 1D: Two-way ANOVA analysis of the numbers of AUC data points listed in the bar charts comparing the calcium response of CHO cells perforated by 0.01% saponin (Figure 4D).

| **Area under curve (AUC)**  **Source of variation** | **Calcium surge upon perforation** | |
| --- | --- | --- |
|  | **F value** | **p value** |
| **Interaction** | 3.47 | **0.0067** |
| **0.01% saponin** | 10.52 | **<0.0001** |
| **ASIC1a overexpression** | 0.1132 | **0.74** |

Supplementary file 1E: Two-way ANOVA analysis of the numbers of AUC data points listed in the bar charts comparing the calcium response of CHO cells to ultrasound under the PcTx1 treatments (Figure 4E).

| **Area under curve (AUC)**  **Source of variation** | **Calcium response persists despite PcTx1 treatment** | |
| --- | --- | --- |
|  | **F value** | **p value** |
| **Interaction** | 1.44 | 0.15 |
| **Ultrasound stimulation** | 17.02 | **<0.0001** |
| **PcTx1 inhibition** | 8.5 | **<0.0001** |

Supplementary file 1F: Two-way ANOVA analysis of the numbers of AUC data points listed in the bar charts comparing the calcium response of ASIC1a overexpressing CHO cells to ultrasound under the PcTx1 treatments (Figure 4F).

| **Area under curve (AUC)**  **Source of variation** | **PcTx1 inhibition on ASIC1a overexpressing CHO cells calcium response** | |
| --- | --- | --- |
|  | **F value** | **p value** |
| **Interaction** | 4.46 | **<0.0001** |
| **Ultrasound stimulation** | 1.26 | 0.29 |
| **PcTx1 inhibition** | 81.03 | **<0.0001** |

Supplementary file 1G: Two-way ANOVA analysis of the DCX staining positive cell counts in the dentate gyrus of the mice treated for 3 consequtively by either sham control or ultrasound as shown in the Fig. 5A. The brain slices were immuno-stained by DCX antibodies and recorded using confocal microscope. The numbers of DCX+ cells (Figure 6) were quantified by using ImageJ and the numbers were subjected to the 2-way ANOVA analysis as shown in this table.

| **DCX cell count**  **Source of variation** | **Dentate gyrus** | |
| --- | --- | --- |
|  | **F value** | **p value** |
| **Interaction** | 0.22 | 0.65 |
| **Genotype** | 26.35 | **0.0002** |
| **Ultrasound**  **treatment** | 9.40 | **0.0098** |

Supplementary file 1H: Two-way ANOVA analysis of the p-ERK cell counts collected from the IHC stained brain slices of wildtype mice, *Asic1a^-/-^* mice and *Asic3^-/-^* mice. Mice of all genotypes were randomly assigned to sham treatment group and ultrasound treatment group. The quantification of p-ERK positive cells were performed using ImageJ with setting of threshold and particle sizes that representing the actual staining pattern. The data were plotted as bar charts (Figure 7) and 2-way ANOVA analysis was performed and the results were shown in this table.

| **p-ERK cell count**  **Source of variation** | **Cortex** | | **Hippocampus** | | **Amygdala** | |
| --- | --- | --- | --- | --- | --- | --- |
|  | **F value** | **p value** | **F value** | **p value** | **F value** | **p value** |
| **Interaction** | 6.45 | **0.0037** | 0.92 | 0.41 | 2.29 | 0.11 |
| **Genotype** | 0.71 | 0.497 | 2.78 | 0.07 | 3.07 | 0.06 |
| **Ultrasound**  **treatment** | 13.43 | **0.0007** | 19.66 | **<0.0001** | 6.83 | **0.0124** |
