# Supplementary material for: ASIC1a is required for neuronal activation via low-intensity ultrasound stimulation in mouse brain
Source: eLife. 2021 Sep 27;10:e61660. doi: 10.7554/eLife.61660 (PMC8510583; doi:10.7554/eLife.61660)
Supplement: Transparent reporting form [file elife-61660-transrepform1.docx]

***eLife’s* transparent reporting form**

We encourage authors to provide detailed information *within their submission* to facilitate the interpretation and replication of experiments. Authors can upload supporting documentation to indicate the use of appropriate reporting guidelines for health-related research (see [EQUATOR Network](http://www.equator-network.org/%20)), life science research (see the [BioSharing Information Resource](https://biosharing.org/)), or the [ARRIVE guidelines](http://www.plosbiology.org/article/info:doi/10.1371/journal.pbio.1000412) for reporting work involving animal research. Where applicable, authors should refer to any relevant reporting standards documents in this form.

If you have any questions, please consult our Journal Policies and/or contact us: [editorial@elifesciences.org](mailto:editorial@elifesciences.org).

**Sample-size estimation**

- You should state whether an appropriate sample size was computed when the study was being designed
- You should state the statistical method of sample size computation and any required assumptions
- If no explicit power analysis was used, you should describe how you decided what sample (replicate) size (number) to use

Please outline where this information can be found within the submission (e.g., sections or figure legends), or explain why this information doesn’t apply to your submission:

The statistical analysis performed for mice in this study is student t-test and the minimal sample size requirement equals to 4. We designed the mice experiments according to the “reduce, refine and replace” principle thus minimizing the number of animals used. Therefore, we have performed our quantitative of mice analysis using N=5 for p-ERK and N=4 for Doublecortin staining.

**Replicates**

- You should report how often each experiment was performed
- You should include a definition of biological versus technical replication
- The data obtained should be provided and sufficient information should be provided to indicate the number of independent biological and/or technical replicates
- If you encountered any outliers, you should describe how these were handled
- Criteria for exclusion/inclusion of data should be clearly stated
- High-throughput sequence data should be uploaded before submission, with a private link for reviewers provided (these are available from both GEO and ArrayExpress)

Please outline where this information can be found within the submission (e.g., sections or figure legends), or explain why this information doesn’t apply to your submission:

1. The p-ERK staining of mouse brain slices were reproducibly performed at least in three different experiments.
2. In fact, the total number of animals tested were more than the data presented in this study. There were slices of brains that were damaged during the processes of staining and fixing the slices on top of the glass slides. Therefore, we quantified only the intact brain slices that were at the same depth/ levels showing similar regions or nuclei. (N=5)
3. In the intracellular calcium response of the primary cultured neurons, the findings were reproducibly performed at least in two different experiments.
4. Calcium response was evaluated repeatedly tested in 4/5 different visual fields under the microscope in each experiment. For quantification, 4/5 different cells were selected as region of interest (ROI) for imageJ image stack reslice analysis and the gray level was plotted versus time.
5. In some cases (Figure 2I, Figure 3B, Figure 3E), the maximum gray levels within the ultrasonic stimulated duration (3 seconds) were plotted to compare the mock-treated responses and inhibitor treatments.
6. The Doublecortin staining indicating adult neurogenesis stimulated by ultrasound treatments were performed in two different experiments. (N=4)

**Statistical reporting**

- Statistical analysis methods should be described and justified
- Raw data should be presented in figures whenever informative to do so (typically when N per group is less than 10)
- For each experiment, you should identify the statistical tests used, exact values of N, definitions of center, methods of multiple test correction, and dispersion and precision measures (e.g., mean, median, SD, SEM, confidence intervals; and, for the major substantive results, a measure of effect size (e.g., Pearson's r, Cohen's d)
- Report exact p-values wherever possible alongside the summary statistics and 95% confidence intervals. These should be reported for all key questions and not only when the p-value is less than 0.05.

Please outline where this information can be found within the submission (e.g., sections or figure legends), or explain why this information doesn’t apply to your submission:

1. Student t test was performed for the p-ERK staining of brain slices in Figure 1D, 1G, 1J. We clearly plotted the number of samples (N=5) and p-Value in the graphs.
2. Student t tests were performed for the maximal calcium response upon ultrasound stimulation comparing the non-treated cells and the specific mechano-receptor inhibitions in Figure 2I. The number of samples of each treatments and p-Value were clearly indicated in the graph.
3. Student t tests were performed for the maximal calcium response upon ultrasound stimulation comparing the mock-treated cells and the extracellular or intracellular calcium blockades in Figure 3B. The number of samples of each treatments and p-Value were clearly indicated in the graph.
4. One-way anova were performed for the maximal calcium response upon ultrasound stimulation comparing the mock-treated cells and the cytoskeletal dynamic inhibitor treatments in Figure 3E. The number of samples of each treatments and p-Value were clearly indicated in the graph.
5. Student t test was performed for the Doublecortin staining of brain slices in Figure 4B. We clearly plotted the number of samples (N=4) in the graph. p-Value was smaller than 0.01

(For large datasets, or papers with a very large number of statistical tests, you may upload a single table file with tests, Ns, etc., with reference to sections in the manuscript.)

**Group allocation**

- Indicate how samples were allocated into experimental groups (in the case of clinical studies, please specify allocation to treatment method); if randomization was used, please also state if restricted randomization was applied
- Indicate if masking was used during group allocation, data collection and/or data analysis

Please outline where this information can be found within the submission (e.g., sections or figure legends), or explain why this information doesn’t apply to your submission:

1. Mice were allocated randomly; for example, if there were two cages of mice, mice were picked from both cages to group as control while the remaining mice were assigned to be ultrasound stimulated.
2. Controls and stimulated mice are age matched and gender matched.

**Additional data files (“source data”)**

- We encourage you to upload relevant additional data files, such as numerical data that are represented as a graph in a figure, or as a summary table
- Where provided, these should be in the most useful format, and they can be uploaded as “Source data” files linked to a main figure or table
- Include model definition files including the full list of parameters used
- Include code used for data analysis (e.g., R, MatLab)
- Avoid stating that data files are “available upon request”

Please indicate the figures or tables for which source data files have been provided:

1. Movie 1 is the source data file for Supplementary Figure 4B the acoustic streaming pattern.
2. Movie 2 is the source data file for the line graph (gray triangle) of main Figure 2B depicting the calcium response to the condition of 2000mVpp duty factor of 0.05% micropipette ultrasound.
3. Movie 3 is the source data file for the line graph (white rhombus) of main Figure 2B depicting the calcium response to the condition of 700mVpp duty factor of 20% micropipette ultrasound.
4. Supplementary Figure S1 and supplementary S2 are the representative stitched visual fields of p-ERK staining in mouse brain quantified in main Figure 1D, 1G, and 1J.
5. Supplementary Figure 5D-K are the source data for the hill curve in main Figure 2C.
6. Supplementary Figure 6A-F are the source data for the PcTx-1 IC50 in main Figure 3A.
